# Surgery

**Published:** 1905-10-07

**Authors:** 


### SURGERY.

### FRACTURES.

*(Continued from page 469.)*

**Injuries of Lower End of Radius.**—To determine the presence of a Colles' fracture of the radius, prolonged and often painful manipulations are to be strongly deprecated. When the radial styloid process, which normally is below the level of that of the ulna, is found to have receded to a level or above this, a fracture can be often diagnosed, but it must be remembered that a Colles' fracture occasionally occurs in its mildest degrees without any such external sign. An *x*-ray picture taken laterally and antero-posteriorly will clearly demonstrate the presence of the injury. In this connection we would draw attention to Dr. Andrew Fullerton's excellent brochure [6] on Colles' fracture and other fractures and disjunctions at the lower end of the radius. From his considerable experience, and with the aid of skiagraphy, he has been enabled to throw some fresh light on certain disputed questions with regard to the mechanism and complications of these common lesions. He protests against the old practice of non-reduction and describes a method of treating Colles's fracture by manipulation under an anæsthetic, and the application of a new and simple form of appliance by an anterior splint or by anterior and dorsal splints, which he describes. He believes that deformity should not result, and that there should be complete restoration of the functions of the hand and forearm.

**Fractures of the Carpus.**—Instances of fracture of the scaphoid bone have been frequently recorded of late since skiagraphy has come into general use. One of the most interesting is that related by Dr. Raymond Russ,[7] and complicated with habitual dislocation of one of the fragments. It occurred in a labourer, aged 32, who presented himself for another slight injury. On the anterior surface of the wrist and directly over the scaphoid there was a small bony prominence about the size of a pea; this could be reduced with slight force when the hand was flexed upon the wrist, but remained displaced in all other positions of the hand. A skiagraph showed transverse fracture of the scaphoid. The displaced fragment adjoined the os magnum and semilunar. There was only slight "grinding" in the wrist at times and no pain. The injury had been caused by a fall three years before when attempting to get on a street car. For a complete exposition of the subject of fracture of the carpal scaphoid we would refer the reader to Drs. E. A. Codman and H. M. Chase's article on this form of injury.[8] They cite the following as a typical history:—A patient, usually a male 25 to 35 years of age, has fallen on his extended wrist, as in the Colles' fracture, and thinks he has sprained his wrist. For a few days there is severe pain, tenderness, and inability to use his hand. He is able gradually to resume his work, but with the continuance of some soreness, tenderness and weakness. The active and passive movements of the wrist joint are limited, and passive movement is accompanied by most characteristic muscle spasm, very similar to that seen in tuberculous joints. There is slight thickening or swelling over the radial half of the wrist joint and tenderness on pressure over the anatomical snuff-box. The *x*-ray shows the transverse fracture of the scaphoid when a good view of the bone at right angles to its long axis is obtained, otherwise it is extremely apt to be deceptive. These authors relate a long series of carefully recorded cases; they advise operation by excision of the fragment after the failure of fixation for four to eight weeks.

**Lower Extremity.**—*Fracture of the Neck of the Femur.*—Too little attention has been given by surgeons to the treatment of fractures of the neck of the femur, yet these form a very important class of injury. Putting aside a number of instances that succumb from such complications as shock, alcoholism, pneumonia, nephritis, exhaustion, etc., there are many in which more efficient and active measures than at present in vogue would probably result in lessening the deformity and prevent the function of the limbs being permanently impaired. Treatment must necessarily be different, according to age, whether in youth or between 20 and 50, or 60 and old age. In childhood operative interference should seldom be required, and in old age is quite out of the question, but in adults better results than those hitherto obtained should not be despaired of. In children proper apposition and union can often be effected by means of traction and the application of the long outside splint, with extension, or by the application of a casing of plaster of Paris. Dr. Royal Whitman[9] advocates in youths and adults a new method of reduction, which certainly should be found most efficacious in many cases, whether the fracture be complete or incomplete. In intracapsular fracture, the patient being anæsthetised, he first flexes the thigh somewhat to reduce the distortion due to the upward and forward displacement of the proximal fragment of the neck. The outward rotation is then corrected, and finally the limb is drawn down by direct traction to its normal length, as proved by measurement, and, lastly, abducted. The latter movement is effected by raising the pelvis upon a sacral support of the Lorenz model, and the limb gently abducted until the normal resistance is encountered. Then under traction, the joint being supported by the hands of the operator, the limb is slowly abducted to the normal limit, 45°. The pelvis is prevented from tilting by simultaneously abducting the sound limb to the same degree. In this attitude a plaster of Paris support is applied. It would appear that a very exact adjustment of the plaster apparatus is essential. In this connection it might be said, at any rate as far as this country is concerned, that surgeons are not such adepts in applying plaster of Paris as they were some years ago. In Germany and America they are ahead of us in this respect. Dr. Whitman advocates open operation in children and young patients where the injury has involved the articulating extremity of the femur. The results of operative treatment, according to Dr. de Forest Willard,[10] do not give perfect results; he inclines to the operation of simply cutting down on the great trochanter, and driving a nail or peg through the neck into the hip, being careful not to perforate the cartilage or go into the joint surface. Whitman opens the joint by the antero-lateral incision on the outer side of the tensor vaginæ femoris; after incising the capsule, a long sharp drill is pushed through the trochanter and the neck into the inner fragment, the outer end being then driven beneath the skin to guard against infection. Without expressing any opinion as to these methods it is to be hoped that in the future the treatment of these fractures will be as successful as that of fractures in other situations.

*Compound Separation of the Lower Epiphysis of the Femur.*—Instances of this injury are always of such magnitude that a brief allusion to two recently related by Dr. G. G. Cottam[11] and successfully treated are worthy of record. They occurred in boys aged respectively 6 years and 9 months and 5 years and 9 months. In both, the diaphysis of the femur protruded through the skin on the outer side of the knee, penetrating the biceps muscle, but the popliteal vessels and nerves were uninjured. In the first case three centimetres of the femoral end were re-sected and the drill and wire employed to secure continued apposition of the fragments—the entire limb was put up in full extension in plaster of Paris. In six weeks the wound was healed, and at the nineteenth week the joint function fully restored, the boy being able to walk without any perceptible limp. However, five and a half years later, as the boy's growth proceeded it was noticed that the knee was becoming "knocked." On skiagraphic examination this condition of bowing with the concavity outwards was found to be entirely due to the continued development of the inner part of the shaft, while the growth of the outer part was arrested. It was proposed to remedy the deformity by osteotomy. In the second case reduction was easily effected, and a piece of bone which had been broken off the anterior surface of the lower end of the shaft removed. The fragment was found broken into several pieces. The limb was put up on an inclined plane, and at the end of the fifth week the knee alone fixed in plaster of paris in a straight position—at the end of seven weeks the soft parts were nearly healed, union of the bone appearing to be normal.

[6] Brit. Med. Jour., June 24, 1905. [7] Annals of Surg., February 1905, p. 265. [8] Ibid., March 1905, p. 321. [9] New York Med. Rec., April 20, 1905, p. 677. [10] Ibid., p. 678. [11] New York Med. Rec., April 1, 1905, p. 487.

(*To be concluded.*)

